# Effects of Soils and Irrigation Volume on Maize Yield, Irrigation Water Productivity, and Nitrogen Uptake

**DOI:** 10.1038/s41598-019-41447-z

**Published:** 2019-05-23

**Authors:** Jing Fang, Yongzhong Su

**Affiliations:** Northwest Institute of Eco-Environment and Resources, Chinese Academy of Sciences/Key Laboratory of Eco-Hydrology in Inland River Basin, Chinese Academy of Sciences, Lanzhou, 730000 China

**Keywords:** Solid Earth sciences, Geophysics

## Abstract

In the newly cultivated oases of northwestern China, the soil properties of farmlands with different cultivation periods show a high degree of spatial heterogeneity on the field scale. However, the irrigation water allocation at the irrigation district scale was based mainly on cultivated area but soil conditions were not considered, which resulted in the shortage or super abound of the irrigation water in the farmlands with different soils. A deeper understanding of the effects of soils on crop IWP and irrigation water requirement is an essential prerequisite to accurately assessing regional irrigation water needs and water-saving potential. Therefore, measurements were taken of the yield, irrigation water productivity (IWP), and nitrogen (N) uptake of maize grown in sandy soils (S1, S2), loamy sand soil (S3), and sandy loam soils (S4, S5) and subjected to three irrigation treatments (full, medium, and low irrigation) in an arid oasis farming system in northwestern China. The results show that the highest yield was obtained under full irrigation in sandy loam. Medium and low irrigation reduced the maize yield by 12.5–21.8% and 13.5–20.6%, respectively, relative to full irrigation, with the greatest decrease in sandy loam. Maize IWP ranged from 1.06–1.20 kg m^−3^ for sand to 2.27–2.58 kg m^−3^ for sandy loam and was influenced by soil properties but not irrigation treatments. Soil properties also influenced crop N uptake, with sandy loam having a significantly higher such uptake than loamy sand or sand. Under a conventional flooding irrigation pattern, reduced irrigation does not appear to increase IWP in well-drained sandy soils. Crop irrigation water requirement and IWP were mainly influenced by soil texture and fertility. Soil management to improving water productivity should be addressed. In agricultural water management, reasonable irrigation water allocation based on soil conditions should be considered.

## Introduction

The ongoing agricultural exploitation and extension of oases toward the deserts in the arid region of northwestern China has considerably enlarged the region’s arable area in the past 50 years. However, it has also aggravated the shortage of water resources and resulted in the over-exploitation of groundwater resources. The use of water by agricultural production has also resulted in ecological deterioration^1^. After the conversion of deserts into irrigated farmlands, crop production becomes heavily reliant on a high input of chemical fertilizers and excessive flooding irrigation due to the very low fertility levels and sandy texture of desert soils^2,3^, which results in poor water and fertilizer use efficiencies, nitrogen (N) leaching loss, and N pollution of the groundwater^2,4^. The adoption of more rational irrigation and fertilization patterns and the development of methods to increase soil fertility and improve water and fertilizer use efficiencies have thus become important aspects of the sustainable agricultural management of newly cultivated oases^5^.

The Heihe River Basin in the arid region of northwestern China is a hotspot for eco-hydrology integration research^6^. Assessment of the crop water requirements and water-saving potential of the oases in the middle of this basin was one of the core elements of the Heihe Eco-Hydrology Integration Research Project^7^. Most of the studies in recent years fall into one of two categories. The first is research dealing with the water requirements^8^ and water balance^9^ of the oasis ecosystem, estimation of the irrigation needs of various crops^10^, and estimation of evapotranspiration by remote sensing on the regional scale. The aim of studies in this category is to determine the reasonable allocation of water resources in ecology and agriculture and in the middle and lower reaches of the inland river basin. The second category of research focuses on the investigation and development of water-saving irrigation technologies such as non-full irrigation, regulated deficit irrigation, and alternative partial root zone irrigation, and on the optimization of agricultural management, including balanced fertilization, plastic film and straw mulching, reduced tillage, and more suitable crop rotation^3,11,12^. The objective is to improve crop IWP on the field scale in the oasis agroecosystem. However, the effects of soil types or soil properties, including soil texture, on crop irrigation water requirements and IWP have generally been neglected. Because soil texture determines the soil water-holding capacity, infiltration, water distribution in the soil profile and transfer pattern, and water retention time in the soil^13^, there exists significant difference in crop irrigation water requirement between different soils with different texture. In the newly cultivated oases of northwestern China, the soil properties of farmlands with different cultivation periods show a high degree of spatial heterogeneity on the field scale^2^. However, the irrigation water allocation at the irrigation district scale was based mainly on cultivated area but soil conditions were not considered, which resulted in the shortage or superabound of the irrigation water in the farmlands with different soils. Therefore, a deeper understanding of the effects of soils on crop IWP and irrigation water requirement is an essential prerequisite to accurately assessing regional irrigation water needs and water-saving potential. Such an understanding can also serve as the basis for implementing more reasonable irrigation water allocation and soil and nutrient practices. The objective of the study reported herein was thus to investigate the effects of different soils and irrigation volume on maize yield, NUP, and IWP.

## Results

### Total irrigation requirements for different soil textures

In this study, the irrigation amount at each irrigation time was controlled, and each soil was treated to full irrigation and water-saving irrigation (medium and low irrigations). However, the irrigation times differed by soil type. Because the determination of irrigation time depended on the occurrence of maize leaf wilting and soil moisture observation, the total irrigation volume needed to be considered as the actual irrigation requirement. Tables 1 and 2 shows that the sand soils required at least eight times the irrigation that the other soil types did, for a total irrigation amount of 7200–9600 m^3^ ha^−1^ during the maize growing season. The sandy loam soil required full irrigation five times during the growing season, for a total irrigation quota of 4800 m^3^ ha^−1^, to maintain normal maize growth.

**Table 1:** Soil physical and chemical properties.

Soil type	Soil layer	Sand	Silt	Clay	Bulk density	Organic matter	Total N	Total P	Total K	Available N	Olsen P	Available K	CEC	pH	EC
		%	%	%	g cm^−3^	kg^−1^	g kg^−1^	g kg^−1^	g kg^−1^	mg kg^−1^	mg kg^−1^	mg kg^−1^	cmol kg^−1^	1:2.5	Μs cm^−1^
S1 Sand	0–20 cm	83.1	7.4	9.6	1.60	5.03	0.38	0.98	12	29.3	33.5	100	4.87	8.9	238
	20–40 cm	87.3	5.2	7.4	1.55	3.54	0.23	0.71	12	19.9	10.7	70	4.04	9.1	212
	40–60 cm	89.8	3.8	6.4	1.55	2.43	0.14	0.65	12	17.2	3.7	60	3.49	9.0	211
	60–80 cm	91.3	2.8	5.8	1.60	2.02	0.12	0.62	12	14.2	2.6	60	2.56	9.1	199
	80–100 cm	91.7	2.6	5.7	1.62	1.78	0.12	0.62	12	10.5	2.0	60	2.60	9.1	196
S2 Sand	0–20 cm	80.1	8.9	11.0	1.49	5.90	0.33	1.00	12	31.9	39.8	90	5.33	8.9	240
	20–40 cm	90.6	5.1	4.3	1.53	3.83	0.21	0.72	12	23.0	6.9	60	4.32	9.1	199
	40–60 cm	91.6	2.4	6.0	1.58	1.78	0.20	0.62	12	17.3	2.0	60	3.31	9.1	193
	60–80 cm	92.5	1.9	5.6	1.63	1.74	0.10	0.66	13	14.3	2.6	60	2.80	9.2	192
	80–100 cm	92.0	2.2	5.8	1.62	1.66	0.10	0.60	12	12.5	2.0	60	2.80	9.2	199
S3 Loamy Sand	0–20 cm	65.5	18.2	16.3	1.49	7.62	0.50	1.08	12	48.7	28.2	110	6.62	8.6	346
	20–40 cm	76.7	11.5	11.8	1.55	5.74	0.40	0.96	12	36.4	30.9	100	5.24	8.7	248
	40–60 cm	87.6	4.9	7.5	1.55	3.34	0.08	0.64	12	15.8	2.3	80	2.94	8.9	190
	60–80 cm	91.8	2.3	5.8	1.56	2.32	0.10	0.52	13	13.6	3.6	80	2.60	9.0	175
	80–100 cm	92.3	2.1	5.6	1.56	1.8	0.08	0.6	12	14.2	2.5	60	2.45	9.1	177
S4 Sandy loam	0–20 cm	57.8	24.4	17.8	1.39	9.99	0.76	1.36	13	61.1	33.9	150	9.37	8.5	314
	20–40 cm	55.4	26.3	18.3	1.44	8.18	0.59	1.12	13	48.8	32.9	130	7.63	8.6	306
	40–60 cm	72.0	14.7	13.3	1.45	7.12	0.43	1.04	12	34.3	9.6	90	6.62	8.8	247
	60–80 cm	85.1	5.5	9.4	1.55	3.21	0.20	0.85	12	28.8	5.8	60	4.62	9.0	192
	80–100 cm	90.8	2.6	6.7	1.56	2.82	0.14	0.86	12	17.2	4.2	60	3.32	9.0	207
S5 Sandy loam	0–20 cm	54.1	27.7	18.2	1.39	11.49	0.85	1.37	13	67.9	33.2	160	9.19	8.5	385
	20–40 cm	50.6	27.7	21.7	1.44	8.87	0.65	1.21	13	62.1	15.6	150	7.81	8.5	364
	40–60 cm	55.1	26.7	18.2	1.49	7.48	0.59	1.16	13	50.4	7.4	140	7.72	8.5	348
	60–80 cm	76.5	11.5	12.0	1.55	6.52	0.30	1.08	13	30.5	5.8	130	5.6	8.8	254
	80–100 cm	90.3	3.0	6.7	1.56	2.56	0.15	0.65	12	18.2	2.6	80	4.32	9.0	172

**Table 2:** Total irrigation volume in the maize growth period in different soil types.

Soil type	Irrigation treatment						
	Irrigation volume at single time, m^3^ ha^−1^			Irrigation time	Total irrigation volume, m^3^ ha^−1^		
	I1	I2	I3		I1	I2	I3
S1	1200	1050	900	8	9600	8550	7500
S2	1200	1050	900	8	9600	8550	7500
S3	1200	1050	900	7	8400	7500	6600
S4	1200	1050	900	6	7200	6450	5700
S5	1200	1050	900	5	6000	5400	4800

### Change in soil water storage

The initial soil water content in the 0–100-cm soil profiles differed significantly among the soil types, with the lowest (99 mm) and highest values (167.5 mm) observed in the sand and sandy loam soils, respectively. No significant differences in soil moisture were observed in the 100–200-cm profiles between soil types because they were of the same texture. After the harvest, the average soil water content in the 0–100-cm soil profiles ranged from 64.3 mm to 104.2 mm across the different irrigation volumes. Soil moisture was significantly higher in S5 than in the other soils. However, soil moisture in the 100–200-cm profile was significantly higher in the sand soils than in the loamy sand and sandy loam soils. No significant differences were found in the soil water content of the 0–100-cm and 100–200-cm profiles between the irrigation treatments for each soil type. In comparison with the initial value before sowing, the soil water content was reduced by 23.4–67.3 mm at the 0–100-cm layer after harvesting. In the 100–200-cm profile, no change in soil moisture was found in the sand soils, whereas a 35.0–35.9-mm reduction, on average, was found in the loamy sand and sandy loam soils (Table 3).

**Table 3:** Soil water storage in the 0–100-cm and 100–200-cm layers before sowing and after harvesting (mm).

Soil	Before sowing, April 20		After harvesting, September 25					
	0–100 cm	100–200 cm	0–100 cm			100–200 cm		
			I1	I2	I3	I1	I2	I3
S1	100.4 c	111.4 a	62.4 b	64.6 b	65.9 b	97.3 a	112.2 a	110.7 a
S2	99.0 c	103.7 a	77.3 b	77.0 b	72.4 b	104.8 a	97.9 a	114.6 a
S3	110.3 b	118.2 a	65.9 b	85.2 b	73.0 b	78.1 b	81.2 b	87.7 b
S4	153.6 a	117.3 a	96.8 b	94.7 b	67.5 b	82.1 b	75.1 b	89.2 b
S5	167.5 a	116.6 a	107.8 a	112.0 a	92.9 a	80.2 b	82.8 b	81.8 b

Lowercase show the significant differences (*p* < 0.05) between soil ty.

### Effects of irrigation volume and soil properties on maize yield and IWP

The maize grain yield was higher under the full irrigation treatment than under the medium and low irrigation treatments for all soil types, and the differences were significant with the exception of S1. However, no significant differences were found between the straw biomass and root biomass for the three irrigation treatments and various soil types. Relative to full irrigation, the total irrigation volumes of the medium and low irrigation treatments fell by 12.5% and 25%, respectively, and the maize grain yields decreased by 12.5% (S1), −21.8% (S5), 13.5% (S1), and −28.0% (S4). Irrigation treatment had a significant effect on the maize thousand-grain weight for all the soil types (Table 4).

**Table 4:** Effects of irrigation treatments and soil types on maize grain yield, straw and root biomass, thousand-grain weight, and irrigation water productivity (values are mean ± SD).

Soil types	Irrigation	Grain yield	Straw biomass	Root biomass	Thousand-grain weight	IWP
	treatment	kg ha^−1^	kg ha^−1^	kg ha^−1^	g	kg m^−3^
S1 Sand	I1	10223 ± 443 a	4925 ± 334 a	571 ± 305 a	328.9 ± 5.2 a	1.07 ± 0.05 a
	I2	9086 ± 727 a	4424 ± 1546 a	350 ± 71 a	291.3 ± 22.3 b	1.06 ± 0.09 a
	I3	9008 ± 678 a	5496 ± 1675 a	411 ± 140 a	299.3 ± 3.4 b	1.20 ± 0.09 a
S2 Sand	I1	10871 ± 537 a	6201 ± 801 a	642 ± 235 a	338.0 ± 4.8 a	1.13 ± 0.06 a
	I2	9280 ± 828 b	5508 ± 247 a	430 ± 124 a	295.4 ± 8.0 b	1.09 ± 0.10 a
	I3	8867 ± 768 b	5496 ± 1355 a	476 ± 41 a	284.0 ± 8.1 b	1.18 ± 0.10 a
S3 Loamy sand	I1	11575 ± 1208 a	6772 ± 548 a	623 ± 223 a	344.4 ± 7.9 a	1.38 ± 0.14 a
	I2	10116 ± 946 ab	5691 ± 509 a	537 ± 109 a	328.0 ± 6.5 b	1.35 ± 0.13 a
	I3	9620 ± 630 b	5563 ± 528 a	600 ± 142a	317.6 ± 8.3 b	1.46 ± 0.10 a
S4 Sandy loam	I1	12172 ± 615 a	7736 ± 382 a	833 ± 104 a	334.4 ± 6.1 a	1.69 ± 0.09 a
	I2	10962 ± 1070 ab	8150 ± 1043 a	665 ± 82 b	340.2 ± 8.0 a	1.70 ± 0.17 a
	I3	9511 ± 418 b	7392 ± 307 a	662 ± 36 b	302.5 ± 13.7 b	1.67 ± 0.07 a
S5 Sandy loam	I1	14897 ± 965 a	8642 ± 342 a	899 ± 108 a	359.9 ± 7.1 a	2.48 ± 0.16 a
	I2	12232 ± 980 b	6863 ± 710 a	817 ± 177 b	348.6 ± 4.6 b	2.27 ± 0.18 a
	I3	12350 ± 1030 b	6973 ± 287 a	827 ± 49 b	320.8 ± 1.2 c	2.57 ± 0.21 a
**Means for soil types**						
S1		9439 ± 802 C	4949 ± 1242 C	444 ± 198 C	306.5 ± 20.7 BC	2.44 ± 0.21 A
S2		9673 ± 1109 C	5735 ± 870 BC	516 ± 170 BC	305.8 ± 25.4 C	1.69 ± 0.10 B
S3		10436 ± 1209 BC	6009 ± 663 B	599 ± 145 B	329.9 ± 13.4 A	1.39 ± 0.12 C
S4		10882 ± 1325 B	7759 ± 342 A	720 ± 139 AB	325.7 ± 24.4 AB	1.13 ± 0.09 D
S5		13160 ± 1662 A	7492 ± 1159 A	848 ± 114 A	343.1 ± 18.0 A	1.11 ± 0.10 D

Lowercase show the significant differences (*p* < 0.05) between the irrigation treatments in each soil type.

Capital letters show the significant differences (*p* < 0.05) across soil types.

Analysis across the irrigation treatments revealed the soil properties to have significant effects on the maize grain yield, straw and root biomass, and thousand-grain weight. The sandy loam with the highest organic matter concentration (S5) produced a significantly higher maize grain yield than the other soils.

**Figure 1:**
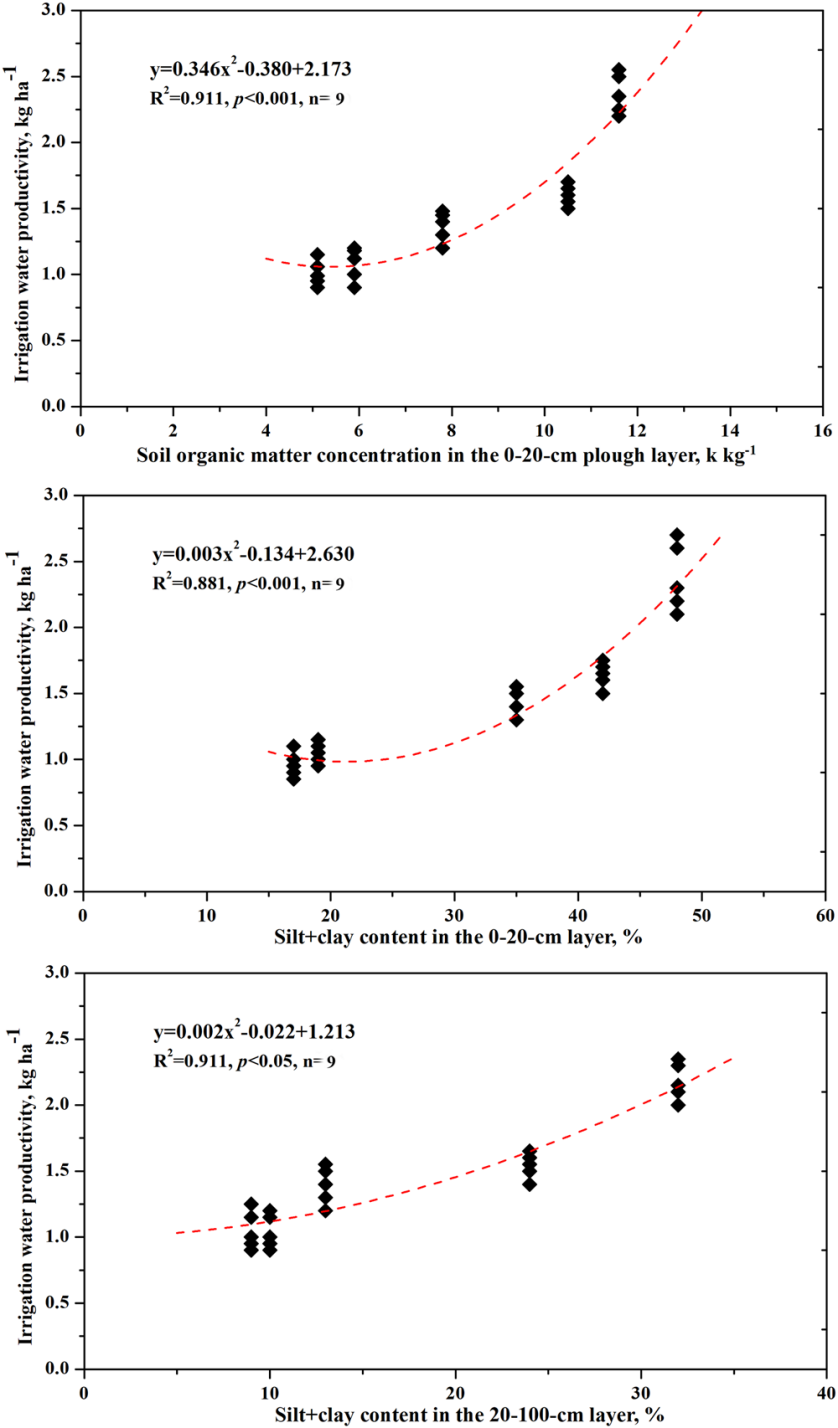
Relationships between irrigation water productivity and soil organic matter concentration in the 0–20-cm plough layer and silt + clay content in the 0–20-cm and 20–100-cm layers.

Maize IWP ranged from 1.06–1.20 kg m^−3^ (S1) to 2.27–2.58 kg m^−3^ (S5), and was influenced by soil type but not by irrigation treatment (Table 4). Regression analysis revealed a significant relationship between IWP and soil organic matter concentration in the 0–20 plough layer and soil silt + clay content in the 0–20-cm and 20–100-cm depths (Fig. 1).

### Effects of irrigation volume and soil properties on maize N uptake and N fertilizer productivity

The type of irrigation treatment was found to have no significant effect on the N content of the maize from the various soil types. Nor did it have a significant effect on the N content of the straw, except that from S1. The N content of the roots was greater under the low and medium irrigation conditions than under full irrigation for S2 and S4 (Table 5). Analysis across the three irrigation treatments revealed a significantly higher grain and root N content in the sandy loam (S4, S5) and loamy sand (S3) compared with the sand soils (S1, S2). The straw N content was higher in the sandy loam and loamy sand soils than in the sand soils, but the difference was not significant.

**Table 5:** Effects of irrigation treatments and soil properties on seed N content, straw and root N content, crop N uptake, Internal N use efficiency and N fertilizer productivity (values are mean ± SD; n = 3).

Soil type	Irrigation	N content in grain	N content in straw	N content in straw	Grain N uptake	Straw N uptake	Root N uptake	Total N uptake	INE	NP
	treatment	g kg ^−1^	g kg ^−1^	g kg ^−1^	kg ha ^−1^	kg ha ^−1^	kg ha ^−1^	kg ha ^−1^	kg kg^−1^	kg kg^−1^
S1	I1	14.5 ± 0.9	7.8 ± 0.4 b	9.3 ± 1.1	149.1 ± 16.2	38.3 ± 0.5 b	5.1 ± 2.1	192.6 ± 15.0	53.2 ± 2.0 a	34.1 ± 1.5
	I2	14.7 ± 0.5 **C**	9.1 ± 1.7 b **A**	14.6 ± 4.1 **B**	133.2 ± 8.0 **C**	38.6 ± 9.1 b **B**	5.2 ± 1.8 **C**	177.0 ± 15.0 **D**	51.4 ± 2.5 ab **A**	30.3 ± 2.4 **C**
	I3	14.8 ± 0.5	11.6 ± 1.1 a	15.1 ± 2.9	133.0 ± 14.3	65.2 ± 23.8 a	6.0 ± 1.4	204.2 ± 34.9	44.7 ± 5.4 b	30.0 ± 2.3
S2	I1	15.3 ± 0.5	8.0 ± 0.9	8.6 ± 3.3 b	166.3 ± 11.4 a	49.2 ± 1.8	5.3 ± 1.8	220.8 ± 12.1	49.3 ± 1.5	36.3 ± 1.8 a
	I2	14.9 ± 1.1 **BC**	9.0 ± 1.2 **A**	13.9 ± 3.1 ab **B**	137.2 ± 3.8 b **C**	49.7 ± 4.6 **B**	6.1 ± 2.4 **C**	193.0 ± 2.5 **D**	48.0 ± 4.0 **AB**	30.9 ± 2.7 b **C**
	I3	15.6 ± 0.3	9.8 ± 1.7	16.0 ± 2.2 a	138.4 ± 13.6 b	52.6 ± 6.1	7.7 ± 2.4	198.8 ± 18.7	44.7 ± 2.0	29.6 ± 2.6 b
S3	I1	16.0 ± 0.7	8.9 ± 0.8	15.0 ± 3.6	185.2 ± 12.7 a	60.5 ± 9.8	8.9 ± 2.4	254.6 ± 22.8	45.5 ± 3.1	38.6 ± 4.0 a
	I2	15.8 ± 0.4 **A**	10.0 ± 1.6 **A**	16.1 ± 2.0 **A**	159.3 ± 10.5 b **B**	56.7 ± 5.7 **B**	9.1 ± 1.1 **B**	225.1 ± 15.6 **C**	44.9 ± 1.7 **BC**	33.7 ± 3.2 b **BC**
	I3	16.4 ± 0.3	11.5 ± 0.6	18.7 ± 1.7	157.3 ± 8.7 b	64.0 ± 13.5	11.1 ± 1.7	232.4 ± 19.5	41.5 ± 2.2	32.1 ± 2.1 b
S4	I1	16.3 ± 2.0	10.2 ± 2.1	15.7 ± 0.4 b	197.8 ± 26.4 a	79.0 ± 16.2	13.1 ± 2.9	289.9 ± 37.1	42.4 ± 5.2	40.6 ± 2.1
	I2	15.9 ± 0.3 **A**	11.7 ± 1.7 **A**	21.8 ± 1.1 a **A**	174.5 ± 14.7 ab **B**	96.3 ± 25.0 **A**	14.5 ± 1.9 **A**	285.2 ± 37.4 **B**	38.6 ± 2.0 **C**	36.5 ± 3.5 **AB**
	I3	16.7 ± 0.5	9.7 ± 2.4	20.0 ± 2.0 a	158.7 ± 3.9 b	71.9 ± 16.5	13.3 ± 1.7	243.8 ± 19.7	39.1 ± 1.8	31.7 ± 1.4
S5	I1	15.2 ± 0.3	10.4 ± 0.3	16.0 ± 5.6	226.8 ± 11.9 a	89.6 ± 14.3	14.7 ± 6.9	331.1 ± 21.9	45.0 ± 1.3	49.7 ± 3.2 a
	I2	15.6 ± 0.8 **B**	12.3 ± 0.3 **A**	20.8 ± 2.0 **A**	190.5 ± 13.4 b **A**	83.9 ± 17.6 **A**	16.8 ± 2.3 **A**	291.1 ± 7.7 **A**	42.1 ± 4.3 **C**	40.8 ± 3.3 b **A**
	I3	15.8 ± 0.3	11.7 ± 0.4	17.8 ± 0.7	194.8 ± 14.3 b	80.8 ± 23.1	14.7 ± 0.9	290.4 ± 30.1	42.6 ± 2.6	41.2 ± 3.5 b

Lowercase show the significant differences (*p* < 0.05) between the irrigation treatments in each soil type.

Capital letters show the significant differences (*p* < 0.05) across soil types.

**Figure 2:**
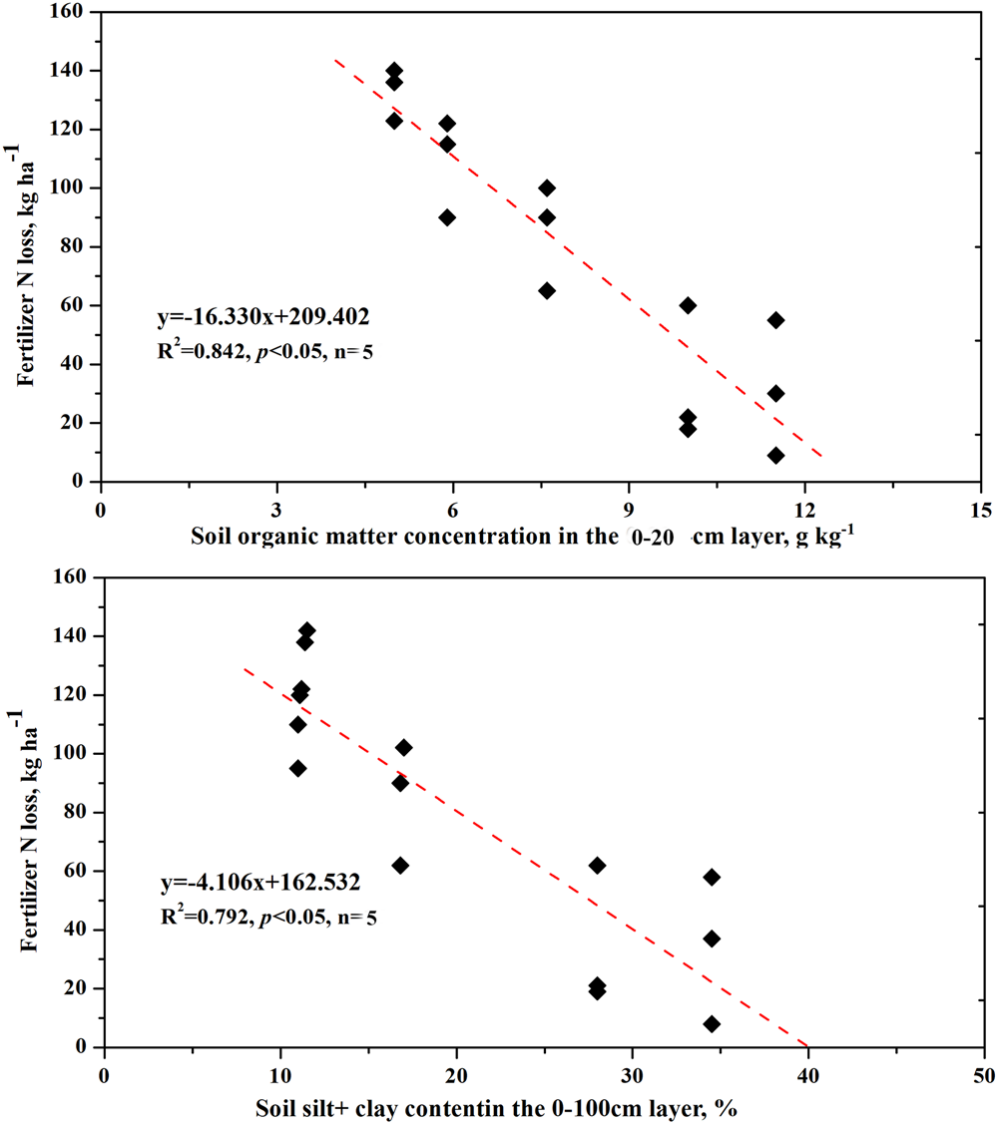
Relationships between fertilizer N loss in the 0–100-cm depth (root zone) and soil organic matter concentration in the 0–20-cm plough layer and silt + clay content in the 0–100 cm layer.

Soil type was found to influence crop NUP, with the sandy loam soil having a significantly higher such uptake (273 kg N ha^−1^ for S4, 304 kg N ha^−1^ for S5, on average) than the loamy sand (237 kg N ha^−1^) and sand (191 kg N ha^−1^ for S1, 204 kg N ha^−1^ for S2). The sandy loam captured more than 90% of the applied N, whereas the loamy sandy and sand captured only 79% and 64–68%, respectively (Table 5). Although no significant differences in total crop NUP were found between irrigation treatments for each soil type, there was an obvious tendency for full irrigation to produce a higher NUP than the other two types of irrigation, except for S1. For the sand soils, the proportion of N in the seeds (N harvest index) was 72.0–72.9%, significantly higher than the 70.5% found in loamy sand and 65–67.1% in sandy loam. The proportion of N in the seeds planted in the same soil type was significantly higher under full irrigation than under either water-saving irrigation, except for those sown in sandy loam (S5). The maximum IEN was obtained for the sand soil, which was significantly higher than that for the sandy loam and loamy sand (Table 2). The NP ranged from 31.5 kg kg^−1^ for S1 to 43.9 kg kg^−1^ for S5, and the NP of the sandy loam was significantly higher than that of the sand. The NP under the full irrigation treatment was significantly higher than that under the low and medium irrigation treatments for each soil type. Regression analysis showed the NP to increase linearly with an increase in soil organic matter, total N concentration, and silt + clay content (Fig. 2).

### Effects of irrigation volume and soil properties on soil NO3-N accumulation and leaching in soil profile

The initial NO_3_-N content varied from 54.7 kg ha^−1^ (sand, S2) to 171.2 kg ha^−1^ (sandy loam, S5) in the 0–100-cm soil layer, and from 24.4 kg ha^−1^ (S2) to 38.2 kg ha^−1^ (S4) in the 100–200-cm soil layer, respectively. After the harvest, the mean residual NO_3_-N in the 0–100-cm layer ranged from 33.7 kg ha^−1^ (S1) to 134.6 kg ha^−1^ (S5), which was lower than the initial values for each soil. However, the average residual NO_3_-N in the 100–200-cm soil layer ranged from 40.5 kg ha^−1^ to 51.7 kg ha^−1^, an increase of 8.3–16.5 kg ha^−1^ compared with the initial values. There was a significant difference in initial and residue NO_3_-N storage between the sand, loamy sand, and sandy loam in the 0–100-cm soil layer, but not in the 100–200-cm layer. No significant difference was found in the residue NO_3_-N amount of the 0–100-cm and 100–200-cm soil layers between the irrigation treatments for each soil type (Table 6).

**Table 6:** NO_3_-N storage (kg ha^−1^) in soil profile (0–100-cm and 100–200-cm layers) before sowing and after harvesting and fertilizer N loss (kg ha^−1^) in the root zone (0–100 cm) (values are mean ± SD; n = 3).

Soil type	Irrigation	Before sowing		After harvesting		Fertilizer
	Treatment	0–100 cm	100–200 cm	0–100 cm	100–200 cm	N loss
S1	I1	58.8	32.8	29.2 ± 1.8 a	43.6 ± 3.7 a	137.0 ± 13.2 a
	I2	58.8	32.8	41.2 ± 17.8 a **C**	45.9 ± 11.1 a **B**	140.6 ± 29.7 a **A**
	I3	58.8	32.8	30.8 ± 8.8 a	47.2 ± 2.6 a	123.8 ± 27.1 a
S2	I1	54.7	24.4	39.6 ± 5.1 a	34.7 ± 8.7 a	94.3 ± 16.0 b
	I2	54.7	24.4	42.2 ± 4.3 a **C**	42.0 ± 7.8 a **B**	119.5 ± 1.8 a **AB**
	I3	54.7	24.4	43.7 ± 8.5 a	44.7 ± 9.3 a	112.2 ± 26.2 a
S3	I1	84.9	35.2	65.8 ± 18.6 a	49.9 ± 14.5 a	64.5 ± 30. 0 b
	I2	84.9	35.2	57.9 ± 7.8 a **B**	48.4 ± 6.4 a **A**	101.9 ± 18.3 a **B**
	I3	84.9	35.2	61.8 ± 7.7 a	56.8 ± 18.2 a	90.7 ± 16.3 a
S4	I1	128.5	38.2	116.8 ± 19.4 a	43. ± 3.0 a	21.8 ± 19.8 b
	I2	128.5	38.2	125.9 ± 9.7 a **A**	52.3 ± 6.3 a **AB**	17.4 ± 38.7 b **C**
	I3	128.5	38.2	125.6 ± 12.1 a	52.8 ± 10.0 a	59.1 ± 14.1 a
S5	I1	171.2	36.8	131.4 ± 26.0 a	54.0 ± 23.0 a	8.7 ± 47.0 c
	I2	171.2	36.8	146.1 ± 16.3 a **A**	44.3 ± 1.4 a **AB**	34.0 ± 12.0 b **D**
	I3	171.2	36.8	126.2 ± 30.4 a	37.0 ± 6.6 a	54.6 ± 53.0 a

Lowercase show the significant differences (*p* < 0.05) between the irrigation treatments in each soil type.

Capital letters show the significant differences (*p* < 0.05) across soil types.

Analysis of the N balance showed the fertilizer N loss to vary from 32.4 kg ha^−1^ to 133.8 kg ha^−1^, on average, and there was a significant difference between the soil types. For the two sandy loam soils, the fertilizer N loss was significant higher under low irrigation than full irrigation. However, no significant differences in fertilizer N loss were found between the irrigation treatments on the sand and loamy sand soils (Table 6). Regression analysis showed a significantly negative linear relationship between fertilizer loss and soil organic matter in the 0–20-cm layer and silt + clay content in the 0–100-cm layer (Fig. 2).

## Discussion

Crop irrigation water productivity (IWP) is influenced by the climate, soil factors, crop cultivar type, irrigation water quality, irrigation technique, and fertilization^14^. Under the same climate condition and irrigation regime, the soil condition becomes a key factor in determining crop IWP^15^. Soil texture determines the soil water-holding capacity, infiltration, water distribution in the soil profile and transfer pattern, and water retention time in the soil, which in turn directly affect the transformation processes between the groundwater and irrigation water, the water balance in the soil-crop system^14^, crop evapotranspiration, and water use efficiency^14,16,17^. In the study reported herein, soil water storage in the 0–100-cm soil profile fell with an increase in sand content (Table 3), which highlights the effect of soil texture on water retention capacity. However, the differences in soil water storage between the irrigation treatments for each soil were not significant, indicating that irrigation volume had little effect on such storage. This result can also be attributed to the high sand content. The sand content of the soils under study increased gradually with an increase in soil depth, with the mean sand content in the 100–200-cm profile measured at nearly 90%^18^. After irrigation, the soil water permeated rapidly into the deep layer of the well-drained soils, which agrees with the result reported in Wang *et al*.^3^ for the same study area. The considerable differences in total irrigation water requirement between soils with different textures and fertility levels may be attributed to the differences in deep percolation. Although drainage amount was not measured in this study, soil water storage in the 0–100 cm depth was higher in sandy loam soil than in sand soils, indicating that greater amount of irrigation water lost through deep percolation in sandy soils.

The mean grain yield varied from 13160 kg ha^−1^ for the sandy loam (S1) to 9434 kg ha^−1^ for the sand (S1), and it increased significantly with an increase in soil organic matter, nutrient concentration, and silt + clay content, thus indicating that soil properties constitute the most important determining factor in crop yield. The highest grain yield was observed under full irrigation for all soils, which suggests that a reduced irrigation volume can result in a significantly reduced crop yield. The low irrigation condition significantly reduced the maize thousand-grain weight compared with full irrigation for each soil, which is one of the reasons for the decreased yield. In the arid oases of northwest China, the conventionally limited irrigation patterns do not support high crop yields in well-drained soils.

In addition to the difference in water-holding capacity between the soils, the difference in maize grain yield was also correlated with the difference in NUP. Razzaghi *et al*.^19^ suggested that soil texture determines the availability of N to plants by influencing both N mineralization and rooting depth and distribution. NO_3_-N leaching loss, N uptake (NUP), and N use efficiency are closely related to soil properties, particularly soil texture^19,20^. Ahmadi *et al*.^20^ investigated the effects of sand, sandy loam, and sandy clay loam soils on potato (*Solanum tuberosum* L.) growth and found differences in yield to be correlated with differences in NUP among the soil types. Razzaghi *et al*.^19^ found that soil with a higher clay content has higher levels of transpiration, crop evapotranspiration, and yield due to the higher uptake of N. In our study, the sandy loam soils contained greater organic matter content than the sand soils, and N mineralization was also generally higher. Furthermore, although we did not measure rooting depth and distribution, the root biomass showed a significant and negative linear relationship with the sand content at the 0–100-cm depth (R^2^ = 0.967). The increased root biomass on sandy loam compared with sand also improved the crop NUP by diffusion, which may constitute an important part of the total uptake^21^. Additionally, in our study, the input of fertilizer N (300 kg ha^−1^) exceeded the crop N demand; therefore, the NUP reflects the influence of the soils’ textural properties on N use efficiency. Although the analysis of N balance and N mineralization did not take it into account, fertilizer N loss through leaching was found to be significantly higher for the sand soils than for the sandy loam soils, showing a significant and negative linear relationship with silt + clay content in the 0–100-cm layer (Fig. 2). Thus, the higher level of N mineralization, deeper root distribution, and lower degree of fertilizer N loss in soils with higher silt and clay contents may explain why crop NUP was greater on the sandy loam than sand soils. The INE was higher for the sand soils than for the sandy loam soils, which suggests more dependence on fertilizer NUP for soils with poor fertility and those that are well drained and sandy.

In the crop production system, water productivity is used to define the relationship between the crops produced and the amount of water involved in that production^13^. In this study, the objective was to analyze irrigation strategies for different soil types. We thus calculated IWP. The amount of water stored in the soil profile at sowing time and the amount of rain falling during the growing season both contribute to seasonal evapotranspiration, but they are not considered in calculating the IWP index. Our results indicate that the average IWP varied from 2.44 kg m^−3^ to 1.11 kg m^−3^ and that it increased with an increase in soil organic matter and silt + clay content (Table 4, Fig. 1). Zwart and Bastiaanssen^22^ reported a global maize IWP range of 1.1–2.7 kg m^−3^. The values in our study fall within this range. However, if the amount of rain that fell during the growing season had been considered, the IWP value for the sand soils would have fallen below the lower limit of the average level. A reduction in irrigation volume did not significantly affect maize IWP in this study because of the reduced yield. The study’s results do not confirm the supposition that a deficit in irrigation can increase crop IWP relative to full irrigation in well-drained soils^1^. However, its results do support the opinion of Katerji *et al*.^23^ that deficit irrigation practices should be avoided in soils with poor total available soil water in the root zone.

The results confirmed that soil texture and fertility determined crop irrigation water requirement and IWP. Therefore, improving the soil structure and soil fertility level should be the most promising ways to increase on-farm crop IWP^15,24^. In agricultural management in the marginal oasis, rational irrigation water allocation should consider soil conditions, and soil management practices should be addressed to improving crop IWP.

## Conclusions

A deeper understanding of the effects of soils on crop IWP and irrigation water requirement is an essential prerequisite to accurately assessing regional irrigation water needs and water-saving potential. The results of this study show that water-saving irrigation (medium and low irrigation treatments) reduces maize yields by 12.0–28.0% compared with the full irrigation of different soils in an arid oasis farming system, although irrigation level appears to have no significant effect on IWP. In this study, soil properties had a clear influence on maize yield, NUP, and IWP. An increase in soil organic matter concentration and silt + clay content in the 0–100-cm profile produced a significant increase in maize yield, NUP, and IWP. In these well-drained soils, soil water and fertilizer N were lost primarily through deep percolation under flooding irrigation. The message for agricultural water management practices is that sandy loam soils with relatively high organic matter content and fine particle fractions produce the highest yield with full irrigation under unlimited water resources conditions. In the soils under study here, water-saving irrigation treatments are not recommended because they result in a considerable loss in yield. However, in the arid region of northwest China, oasis farming systems must reduce their water consumption in the face of the current and expected future lack of water. Therefore, introducing other advanced irrigation techniques such as drip irrigation should be considered. An improvement in soil fertility can result in significant increases in water productivity. From the sustainable agriculture point of view, improved land management is one of the most promising ways to increase on-farm water productivity. Hence, soil management practices that are known to improve soil fertility and soil water storage, such as the use of less tillage, the application of manure, the return of crop residues to the soil, or the conversion of crops into perennial forage, should be considered.

## Materials and Methods

### Study area

The study area was the Linze marginal oasis in the middle reaches of the Heihe River Basin in Gansu Province, northwestern China. This oasis has recently been developed for agricultural production (39°24′ N, 100°21′E; altitude, 1360–1385 m). North of the oasis are the gravel Gobi desert and sand dunes. The region has a typical desert climate and is characterized by cold winters and dry, hot summers, with a mean annual precipitation of 117 mm. Mean annual evaporation is 2,390 mm, and the average annual temperature is 7.6 °C. There are approximately 165 frost-free days per year, and the growing period is from March to October. The groundwater level ranges from 4 to 10 m. The zonal soil in the marginal oasis is made up of calci-orthic aridisols derived from diluvial-alluvial materials, according to Chinese soil taxonomy, and these are equivalent to calciorthids in the U.S. Department of Agriculture’s soil taxonomy classification^25^. Due to the long-term encroachment of drift sand from the Badanjilin Desert and the deposition of aeolian sand, psamments have developed in some areas, leading to sandy land formation. Since the 1960s, these sandy lands have gradually been reclaimed for agriculture use. The soils on newly reclaimed land have very low nutrient concentrations and a loose sandy structure. Increases in cultivation duration and gradual improvements in the physical and chemical properties of various soils have led to obvious gradient changes in their texture, structure, and nutrient levels at the plough layer in different cultivation years^2^.

**Figure 3:**
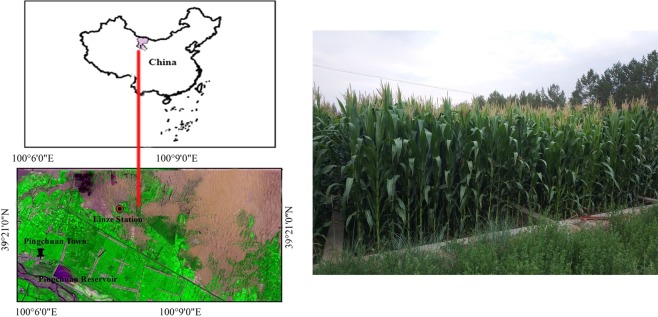
Study area and experimental site. The maps are generated with Arc Map Ver 10.1 (https://www.esri.com/en-us/store/arcgis-desktop).

The major crops grown in the study area are maize (*Zea mays* L.) and spring wheat (*Triticum aestivum* L.), both of which are harvested once annually. In the past decade, seed maize production has become the backbone of agriculture, and most farmlands in the area have now been under a continuous seed maize monoculture for more than five years. Plastic mulching has been commonly practiced for maize cultivation in the past two decades. Recent years have also seen groundwater used for crop production, accounting for more than 50% of total irrigation water. Conventional tillage management is commonly adopted, with fields usually plowed twice before sowing. After harvesting, the stalks of the wheat and maize are removed. Croplands are ploughed using moldboard in the fall. In winter, croplands are irrigated to store soil water, and in spring they are disked before sowing. The amount of fertilizer applied to maize annually has been about 270 to 400 kg^−1^ N ha^−1^, 90 to 150 kg P_2_O_5_ ha^−1^, and 60 to 90 kg K_2_O ha^−1^ in the past 10 years. Farmlands are irrigated 6 to 11 times during the maize growing season each year, depending on the soil conditions^2^ (Fig. 3).

### Experimental layout

Under natural conditions, it is difficult to find fields close to one another that differ in soil type or texture and fertility level. Studies of the effects of soils on crop water use efficiency or IWP are thus generally carried out using the lysimeter method^14,19,23^. In this study, we chose farmlands with different cultivation periods that differed in soil texture and fertility. Soils from these farmlands were then displaced to the irrigation experiment fields at the Linze Inland River Basin Research Station, Chinese Academy of Sciences (39°21′N, 100°07′E; altitude, 1367 m). The soils at 0–100 cm in depth were displaced with a separate layer that was 20 cm in depth. Each soil was displaced to nine experimental plots with an area of 20 m^2^ (4 × 5 m). Following soil displacement, full irrigation was performed at the end of October 2014. The soils at the 0–100-cm depth between each plot were separated with impermeable material (a rubber sheet) to protect against water lateral infiltration, and a 2-m neutron tube was placed in the center of each plot. The experiment was performed in 2015 and 2016. Before sowing in the middle of April of that year, soil samples at the 0–100-cm depth were taken at increments of 20 cm in each plot. The physical and chemical properties of each soil type were measured and are listed in Table 1. Soil property measurements at the 0–20-cm plough layer showed that the soil texture was sand (S1, S2), loamy sand (S3), and sandy loam (S4, S5). The soil organic matter, N, phosphorous (P) and potassium (K) concentrations, and cation-exchange capacity (CEC) increased with a decrease in sand content (Table 1). The soils in the 100-cm depth below were not displaced, and the mean sand content in the 100–200-cm layer was close to 90%^18^. Rainfall in 2015 and 2016 amounted to 118–123 mm, 92–102 mm of it during the maize growing season.

### Irrigation treatment

The irrigation times and total irrigation amount during the crop growing season differed considerably among the soils with different textures due to their different water-holding capacities and leakage characteristics. For reasons of practicality, we designed three irrigation treatments for each soil type: full irrigation (I_1_, 1200 m^3^ ha^−1^ at each irrigation time), medium irrigation (I_2_, 1050 m^3^ ha^−1^ at each irrigation time), and low irrigation (I_3_: 900 m^3^ ha^−1^ at each irrigation time). However, the irrigation times differed for each soil type. The different irrigation interval times meant that the determination of the irrigation time for each soil type depended on the soil moisture. Therefore, the total irrigation amount applied during the maize growth period was calculated by multiplying a single irrigation amount by the number of irrigation times for each treatment of each soil type (Table 2). The irrigation treatments were arranged into a sequential block design with three replicates to manage irrigation conveniently for each soil type. The irrigation amount was controlled by a water meter.

### Field management

Each plot was mulched with four rows of plastic film (0.6 m in width and 5 m in length). Each row of plastic film sowed two rows of maize, and the inter- and intra-row spacing was 0.40 m and 0.25 m, respectively. The maize cultivar was Zhangdan 397. The fertilizer application rate for each soil type was 300 kg N ha^−1^, 150 kg P_2_O_5_ ha^−1^. The N and P were applied as urea and diammonium phosphate, respectively. The overall P fertilizer and one third of the N fertilizer were applied as basic fertilizers during sowing. The other two thirds of the N fertilizer was top-dressed twice: during the maize elongation stage and booting stage. The maize was sown on April 20–22, and harvested on September 20–25. The management practices were conducted according to local agronomic practices unless otherwise indicated.

### Measurements, sampling, and sample analysis

Before sowing, 24 h before each irrigation time, and after harvesting, the soil moisture at a depth of 0–200 cm was measured using a neutron instrument. During the maize growing season, before sowing, at the maize elongation and booting stages, and after harvesting, soil samples were taken at increments of 20 cm within 200 cm from each plot using an auger with a diameter of 3.6 cm. A part of each sample was weighed and then dried to a constant weight at 105 °C to gravimetrically determine the soil moisture content. Another part of each sample was stored in a plastic bag and analyzed to determine the NO_3_^−^N content.

The grain and straw yields of all the treatments were determined from three replications at the end of the growth period by harvesting the maize from a 12 m^2^ area at the center of each plot. At harvest time, all plants within the 12 m^2^ area of each plot were cut near the soil surface, with the roots left in the field. The roots of five plants were excavated to determine the root biomass. The dry matter content of the grain, straw (stalks and leaves), and roots were determined by drying the sub-samples at 65 °C to a constant weight. The samples were then milled to a fine powder with a particle size of <0.2 mm to determine the total N content of the plant (plant N) and to calculate its total N uptake (NUP). The total N content in the grain, straw, and roots of the sub-samples was measured with a C/N elemental analyzer (Elemetar Macro Cube, Germany). The NUP by plant N was estimated by multiplying the grain, straw, and root dry matter weights by their N concentrations.

### Data analysis

N fertilizer productivity and maize irrigation productivity were analyzed using the following:

$${\rm{Maize}}\,{\rm{IWP}}={\mathrm{GYi}/I}_{{\rm{i}}}$$

$${\rm{Internal}}\,{\rm{N}}\,{\rm{use}}\,{\rm{efficiency}}\,({\rm{INE}})={\mathrm{GYi}/\mathrm{NUP}}_{{\rm{i}}}$$

$${\rm{N}}\,{\rm{fertilizer}}\,{\rm{productivity}}\,({\rm{NP}})={\mathrm{GYi}/N}_{{\rm{i}}}$$

where GYi is the grain yield (kg ha^−1^), I_i_ is irrigation volume (m^3^ ha^−1^), NUPi (kg ha^−1^) is N uptake by the crop, and N_i_ (kg ha^−1^) is the N fertilizer rate (300 kg N ha^−1^)^13,26^.

Losses of fertilizer N were calculated according to a modified version of the equation presented in^1^, where N fertilizer fixation is neglected:

$${\rm{Fertilizer}}\,{\rm{N}}\,{\rm{loss}}={{\rm{N}}}_{{\rm{initial}}}+{{\rm{N}}}_{{\rm{input}}}-{{\rm{N}}}_{{\rm{uptake}}}-{{\rm{N}}}_{{\rm{residual}}},$$

where Ninitial is the initial soil NO_3_-N in the 0–100-cm soil profiles, N_input_ is the N application rate (300 kg N ha^−1^), N_uptake_ is the NUP by the plant, and N_residual_ is the residual NO_3_-N in the 0–100-cm soil profiles. In this N balance equation, the other NUPs, such as irrigation water NUP, are neglected. NO_3_-N storage below the 100-cm soil depth was not included in the N balance equation because maize roots are distributed at a depth of 0–100 cm, particularly in sandy soils. In addition, the NH_4_-N throughout the soil profile was not considered due to the relatively few changes in NH_4_-N content between seasons^26^. Fertilizer N loss was considered primarily as NO_3_-N leaching, as other N losses via denitrification and volatilization are relatively low in arid desert oasis soil^27^.

### Statistical analyses

Two-way analyses of variance (ANOVA) were used to test the effects of the three irrigation treatments and the five soils on grain yields and biomass, crop NUP and INE, and maize IWP. Pairs of mean values were compared by the least significant difference at the 5% and 1% levels using the SPSS software package. The relationships between soil properties, IWP, and fertilizer N loss were evaluated by linear or curvilinear regressions.
